# Computational Screening of Glyme-Based Room-Temperature Aluminum Plating Solutions

**DOI:** 10.1021/acsomega.4c09455

**Published:** 2025-01-10

**Authors:** Tomoya Kanno, Tsubasa Otsuki, Norio Takenaka, Atsushi Kitada

**Affiliations:** Department of Chemical System Engineering, The University of Tokyo, 7-3-1 Hongo, Bunkyo-ku 113-8656, Tokyo, Japan

## Abstract

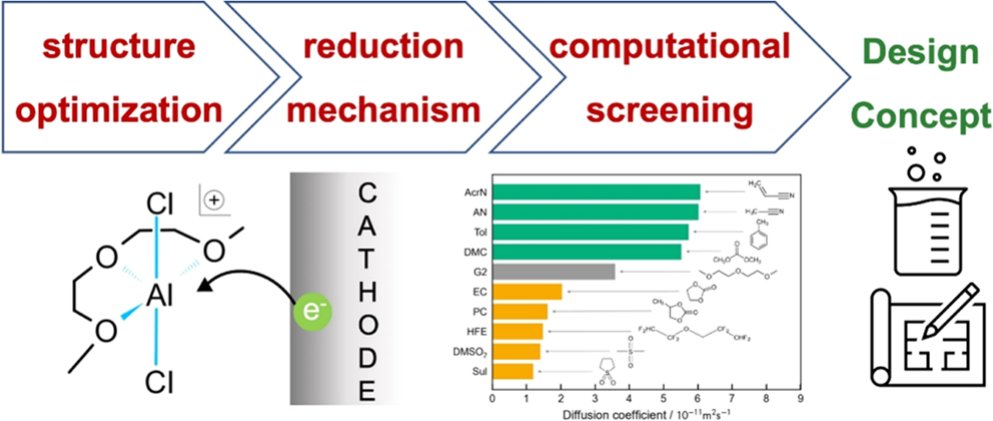

A computational data-driven approach is applied to the design of liquid electrolyte materials for a complex phenomenon, viz. electrochemical deposition. A protocol for the liquid electrolyte material exploration consists of (i) structure optimization, (ii) reduction mechanism elucidation, and (iii) computational screening. A case study is conducted targeting glyme-based room-temperature aluminum (Al) electroplating solutions, where how to achieve a higher plating speed has been an open issue. The determination of stable Al–Cl–glyme complex structures by density functional theory calculations enables the modeling for molecular dynamics simulations of the bulk electrolytes, namely, aluminum chloride (AlCl_3_)–diglyme (G2)–cosolvent system. It is shown that a tridentate coordination of diglyme (G2) to [AlCl_2_]^+^ is robust against desolvation and one-electron reduction, suggesting that the diffusion coefficients of the [AlCl_2_]^+^ cationic complex are an indication of faster plating. Additionally, the calculated diffusion coefficients correlate weakly with the relative permittivity data of cosolvents in their pure state but strongly with viscosity data. This relationship can be used to design Al plating solutions.

## Introduction

Aluminum (Al) metal is an essential material in everyday life, found in structural components, cases, housings, cans, and coins, where its properties such as lightweight, decorative appeal, corrosion resistance, wear resistance, and durability are exploited. Al electroplating or electrodeposition offers the ability to impart these properties to dissimilar material surfaces, thereby enhancing the value and life of industrial products.^1,2^ Electroplating is particularly attractive due to its higher energy efficiency and ease of thickness control compared with methods such as thermal spraying or molten metal deposition. Consequently, various Al electroplating processes are being investigated with a focus on electrolyte formulations.^3−5^ Furthermore, Al electroplating is a bottom-up approach that could directly produce Al films or foils for next-generation current collectors of lithium ion battery,^6,7^ potentially replacing current rolling processes and reducing the rolling costs of the Al ingots. In addition, advances in improved recycling processes are aimed at purifying Al alloy waste, recovering Al purity, and promoting material circulation of Al materials.^8^ Another promising aspect of Al electroplating is the development of next-generation “Al-ion” batteries that utilize aluminum with its low oxidation potential (−1.68 V vs SHE) and high theoretical capacity (8042 mAh cm^–3^) as a negative electrode material.^9−12^ Therefore, material exploration for next-generation Al electroplating can offer various kinds of industrial applications including material circulation and energy storage, not only for surface finishing.

High-temperature aluminum electrolytes, exemplified by alumina-based high-temperature molten salts in practical Hall–Héroult processes, have led to continued interest in electrolytes capable of aluminum plating at medium and low temperatures to prevent undesirable alloying with substrates and impurities. These electrolytes continue to be highlighted for their applications in plating, recycling, and battery technologies. Over the past decades, various electrolytes, including ionic liquids^13−21^ and organic solvents,^22−46^ have been proposed for room-temperature (RT) aluminum plating. In particular, glymes, viz. CH_3_O(CH_2_CH_2_O)_*n*_CH_3_, are readily available industrial surfactant raw materials characterized by low volatility at RT (due to boiling points above 160 °C), which enhances safety as organic solvents for plating.^42−46^ In glyme-based electrolytes, the electrochemically active species are presumed to be [AlCl_2_(glyme)_2_]^+^ cationic complexes, as opposed to [Al_2_Cl_7_]^−^ and [Al(solvent)_3_]^3+^ in many other electrolytes.^42,43,46,47^ Nevertheless, the detailed structure of [AlCl_2_(glyme)_2_]^+^ complexes remains elusive, posing a challenge for advanced electrolyte design.

In the case of ionic liquids and other organic solvents containing aromatic rings and/or long alkyl chains, the addition of aromatic or alkane cosolvents has been shown to improve electrolyte properties based on π–π interactions and van der Waals forces.^13,31,48−50^ However, for glyme-based systems lacking aromatic rings or long alkyl chains, guidelines for cosolvent selection are not straightforward. Combinatorial synthesis and automated experiments are one approach, but experimental screening of different cosolvents without clear guidelines is inefficient and increases the experimental costs.

In contrast, recent efforts have used molecular dynamics (MD) simulations and computational screening based on structure–property calculations to overcome experimental challenges in materials discovery and propose candidate materials for applications such as bioethanol separation, organic solar cells, carbon capture membranes, and ion-conducting solids.^51−55^ To the best of our knowledge, such computational methods have not been applied to screen liquid electrolyte materials with high diffusivity because large time-scale simulation (nanosecond order) is required for reliability and ab initio MD with high computational cost is not available. The development of electrolytes with high ionic conductivity and diffusion coefficients is critical for increasing reaction rates and minimizing ohmic losses in electrochemical systems, thereby promoting sustainability. In this study, we present a computational screening approach for glyme-based room-temperature aluminum plating electrolytes using classical MD simulations, which can deliver long-term diffusive properties (nanosecond order) with a low computational cost, providing insight into cosolvent addition to enhance performance.

## Methods

Density functional theory (DFT) calculations were performed for structural optimization and energy calculations of glyme complexes using the Gaussian 16W suite of electronic structure programs, where the B3PW91 density functional was used for all molecules along with the 6-311 + G(d) basis set. Instead of a vacuum- or gas-phase condition, the solvation model, namely, the SMD method, was used with tetrahydrofuran (THF) as the solvent phase,^56^ since the dielectric constant of glymes is similar to that of THF.

The program used for the MD simulations was AMBER. The initial structure of each molecule is the structure optimized by DFT under the conditions described above. The restrained electrostatic potential (RESP) charge calculated by DFT was used to calculate the charge of each molecule. The cell sizes were adjusted by conducting NPT-MD simulations (1 bar and 298 K) for each electrolyte (see more details in the Supporting Information).

According to previous studies, the neutral solute AlCl_3_ is estimated to dissociate into cationic [AlCl_2_(G2)_2_]^+^ coordinated with glyme and anionic [AlCl_4_]^−^ in equimolar proportions.^46^ Based on the experimental findings, the atomic ratios in the simulation cell were set to [AlCl_2_]^+^/[AlCl_4_]^−^/G2 = 80:80:800 for pure AlCl_3_/G2 solution and [AlCl_2_]^+^/[AlCl_4_]^−^/G2/cosolvent = 80:80:400:400 for a comparison system of G2-cosolvent. Force fields for G2 and cosolvents used general AMBER (GAFF) parameters,^57^ while parameters for [AlCl_4_]^−^ were based on the literature.^58^ Since parameters for [AlCl_2_]^+^ were not reported, new adjustments were made based on DFT calculations of Al complex structure optimizations, specifically the Al–O ether oxygen distances coordinated with glyme, to replicate in MD simulations. Specifically, due to the high covalent nature of the Al–Cl bonds in [AlCl_2_]^+^ and [AlCl_4_]^−^, these complexes fix the Al–Cl bonds and do not consider vibration. This treatment allowed classical MD simulations to be performed at realistic computational speeds, allowing for the calculation of self-diffusion coefficients. In the DFT calculations, it was determined that the average distance between the Al atom of [AlCl_2_]^+^ and the O atom of G2 is 2.01 Å, which guided the adjustment of the force field parameters (Lennard-Jones potential with distance parameter σ and depth parameter ε) for [AlCl_2_]^+^. As shown in Figures S1 and S2, the peak distance of the Al–O radial distribution function (RDF) was 2.01 Å at σ = 0.9 Å, and this parameter set was used in the MD simulations. The force field parameters for [AlCl_2_]^+^ are given in Tables S2 and S3.

The diffusion coefficient *D* of [AlCl_2_]^+^ was determined by the Einstein equation^59^
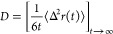
1⟨Δ^2^*r*(*t*)⟩ is calculated from the slope of the mean-square displacement (MSD) of the Al atom in [AlCl_2_]^+^ by performing a least-squares approximation in the range of 100–1000 ps (Figure S3, red line).

## Results and Discussion

**Figure 1:**
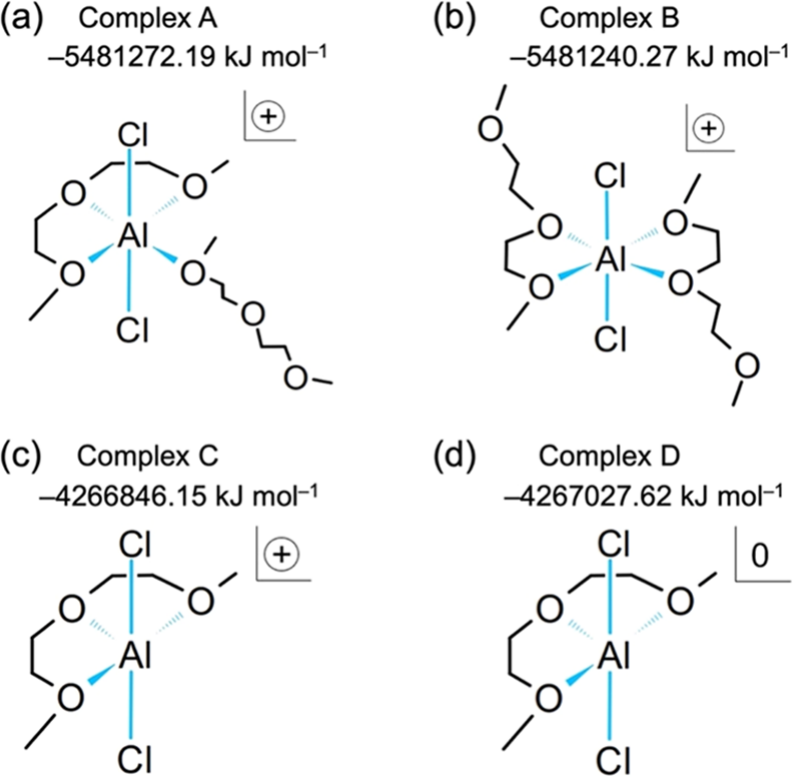
Schematic structures of the Al–Cl–glyme complexes A–D with their calculated energies obtained by the optimization: (a) complex A with one tridentate G2 and one monodentate G2 coordination, (b) complex B with two bidentate G2 coordinations, (c) complex C with a tridentate G2 coordination, and (d) complex D with one tridentate coordination and one-electron reduced state.

First, the structure of the cationic glyme complex was determined by DFT. From previous spectroscopic studies including DFT calculations, it is known that two Cl and four O atoms are coordinated around Al to form [AlCl_2_(glyme)_*n*_]^+^.^42,43,46,47^ Nevertheless, especially for the electrochemically active G2 species, it has been an open question that which of the two possible structures for the cationic complex are stable in the G2 electrolyte,^43^ as shown in Figure 1a,1b: the first is complex A, in which two G2, one tridentate and one monodentate, are bound to [AlCl_2_]^+^; the second is complex B, in which two bidentate G2 are present. We optimized these structures and calculated their energies, showing that complex A has a significantly lower energy of 32 kJ mol^–1^ and is dominant over complex B.

Next, the bond strength of the monodentate G2 ligand was examined. When the monodentate diglyme is removed from complex A, complex C with one G2 tridentate ligand is formed (Figure 1c). Optimization of the structure before and after removal of the monodentate ligand revealed that complex C is stabilized by the bending of the Cl–Al–Cl bond (see Figure S4), resulting in a Gibbs energy of about −5 kJ mol^–1^ for the reaction, complex A → complex C + G2 (Table 1). The negative energy change from complex A to complex C suggests that a spontaneous desolvation reaction may occur with no special treatment such as electrodeposition. However, the energy difference between complex A and complex C is not so large, allowing coexistence of the two complexes in the solutions. Thus, the monodentate diglyme can spontaneously desorb from complex A, with a thermodynamic existence ratio of [complex A]/[complex C] being about 1:7.5 by mol.

**Table 1:** Energy Required to Desolvate One G2 Ligand from Complexes A and C

reaction	energy (kJ mol^–1^)
complex A → complex C + G2	–5.287
complex C → [AlCl_2_]^+^ + G2	553.7

To predict the reduction mechanism, we turned to the one-electron reduced state of complex C. When the cationic complex C receives an electron, the neutral complex D is formed (Figure 1d), and the energy gain from its formation was calculated to be about 181 kJ mol^–1^. Furthermore, as shown in Table 1, the desolvation energy required to desolvate tridentate G2 of complex C is quite high (553.7 kJ mol^–1^). Therefore, it is strongly suggested that in this glyme-based Al plating solution, the one-electron reduction occurs prior to the desolvation of tridentate G2 to form complex D. The fact that the Mulliken charge of the Al atom is about 1 unit smaller after the one-electron transfer also suggests that the Al atom received an electron (Figure S4).

**Figure 2:**
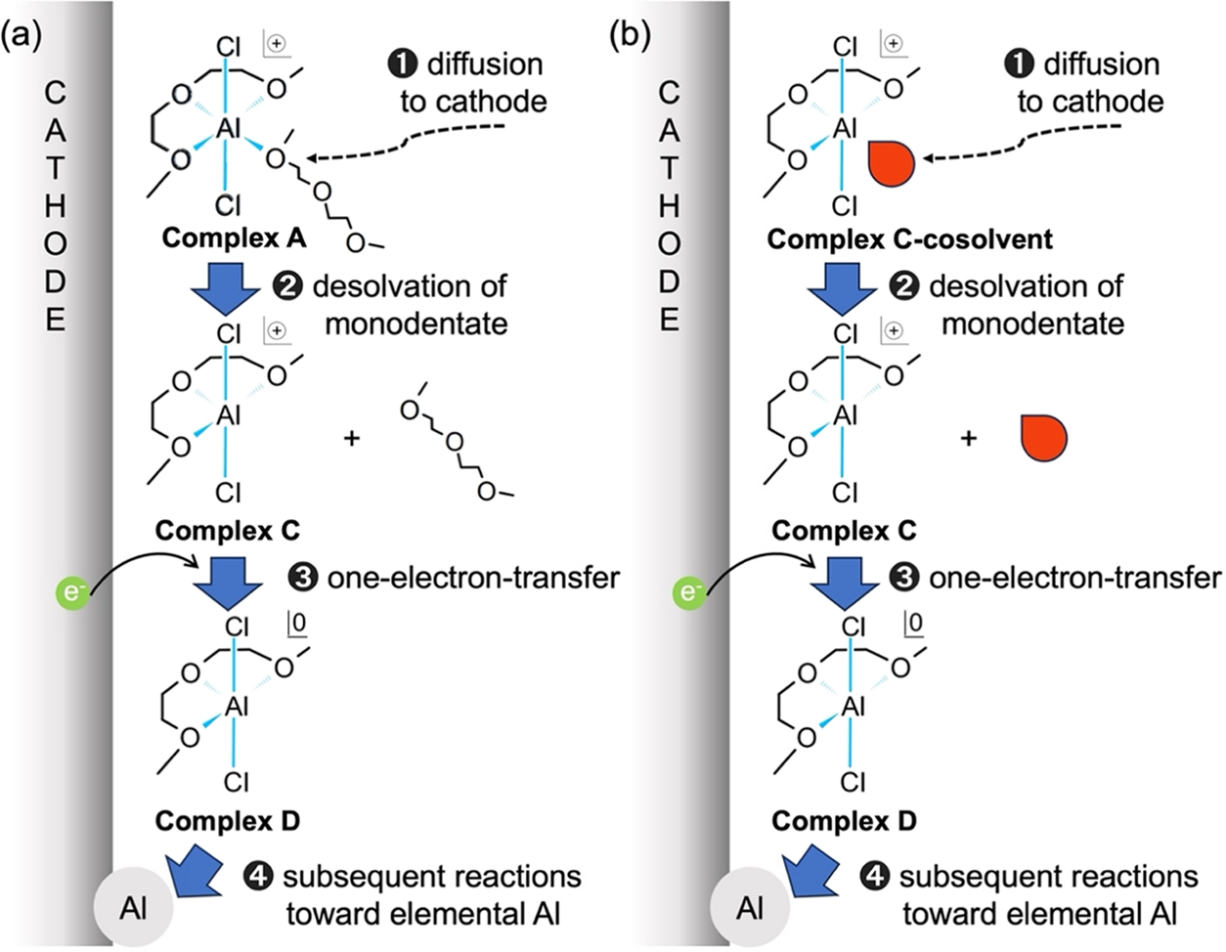
Predicted mechanism of Al electroplating in the (a) G2–AlCl_3_ system and (b) G2–AlCl_3_–cosolvent system. In both cases, diffusion to the cathode initiates the process, followed by desolvation of the monodentate, one-electron transfer, and subsequent reactions leading to Al metal deposition at the cathode.

Based on the above first-principles calculations, we predicted the reduction mechanism of the Al–glyme complex (Figure 2a). First, the glyme complex approaches the cathode by diffusion. Next, the G2 monodentate is desolvated, and complex C is formed. Then, complex C undergoes a one-electron reduction to form complex D. Finally, a subsequent reaction involving desolvation of the G2 tridentate and acceptance of the remaining two electrons occurs, leading to the deposition of elemental Al.

The Al–glyme–cosolvent complexes are then examined in the same manner. The cosolvent used in this study meets the basic requirements for a practical electrolyte, such as viscosity, (electro)chemical stability, and material cost, and is commonly used in the lithium ion battery study.^60^ Specifically, two types of carbonates (ethylene carbonate (EC) and propylene carbonate (PC)), two types of nitriles (acetonitrile (AN) and acrylonitrile (AcrN)), and two sulfone solvents (dimethylsulfone (DMSO_2_) and sulfolane (Sul)) are selected as high dielectric cosolvents. Dimethyl carbonate (DMC), 1,1,2,2-tetrafluoroethyl 2,2,3,3-tetrafluoropropyl ether (hydrofluoroether; HFE), and toluene (Tol) were selected as low dielectric cosolvents. DFT calculations revealed that Tol and HFE are essentially uncoordinated to Al(III), while the other cosolvents are coordinated to complex C as a monodentate. Furthermore, the energy required for desorption of a cosolvent is about 24 kJ mol^–1^ at most (see Table S1), and it can be said that cosolvents are easily desorbed. Therefore, the content of [AlCl_2_(EC)_4_]^+^ in the electrolyte in this study is significantly small where an equimolar amount of G2 and EC is mixed, while the EC complex is detected in a glyme-free AlCl_3_/EC solution.^41^ Based on the above, the cosolvent complexes are considered to have a reduction mechanism similar to that of pure glyme complexes (see Figure 2b).

From the above discussion, it is expected that diffusion plays an important role in the reduction mechanism in the case of a glyme-based Al plating solution. In other words, if the diffusion coefficient of Al–glyme complexes increases with the addition of cosolvents, then the diffusion of the complexes to the cathode will be faster, leading to an increase in electroplating speed. Therefore, considering the diffusion coefficient as an indicator of electroplating speed improvement, MD simulations were performed in the presence and absence of each cosolvent. In the Supporting Information Video, G2 and cosolvent are not shown in the diffusion of the [AlCl_2_]^+^ cationic species for clarity.

**Figure 3:**
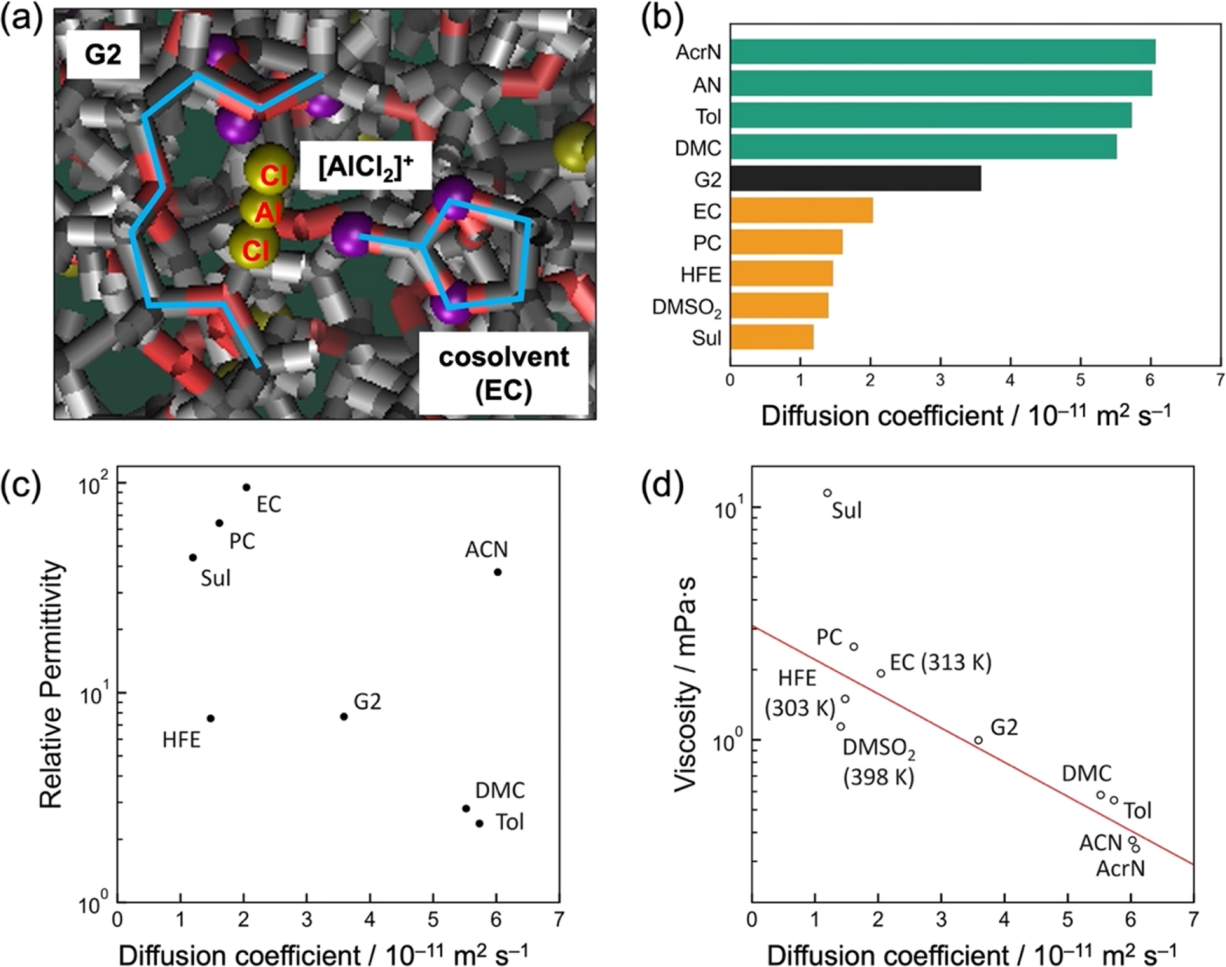
MD simulation results and correlations between the physicochemical properties. (a) A snapshot of the MD simulations for the G2–AlCl_3_–EC system, focusing on [complex C-EC], namely, the complex C [AlCl_2_(G2)]^+^ coordinated by an EC cosolvent, (b) the calculated diffusion coefficients of the [AlCl_2_]^+^ complexes by varying the cosolvent, and (c, d) the literature values of (c) relative permittivity and (d) viscosity for pure cosolvents plotted against the calculated diffusion coefficients.

Figure 3a shows a snapshot of the MD for an electrolyte with EC as a cosolvent. The light blue line on the left is G2, the light blue line on the right is EC, and the yellow gold sphere in the middle is [AlCl_2_]^+^, supporting the DFT calculation results (Table S1), i.e., the tridentate coordination of the oxygen in G2 around the Al atom and the monodentate coordination of the oxygen in EC. Thus, the structures obtained by DFT are well reproduced in classical MD, proving the validity of the force field parameters for [AlCl_2_]^+^ modified in this study. Building the classical force field parameters of [AlCl_2_]^+^ for the first time is of special importance, since the dichloro-aluminum complex forms not only in ethereal electrolytes but also in carbonate ones.^41^ The screening results for the calculated diffusion coefficients of the cationic Al complex in pure glyme-based and cosolvent electrolytes are shown in Figure 3b. Based on the mechanism described earlier, the larger the diffusion coefficient, the faster the diffusion rate of the Al complex approaching the cathode and the faster the deposition rate. Therefore, using diffusion as an indicator, DMC, Tol, AN, and AcrN are considered to be effective cosolvents. Furthermore, while there is no correlation between the calculated diffusion coefficient of the complex and the relative permittivity of the pure cosolvent obtained from handbooks (Figure 3c), there is a high correlation between the calculated diffusion coefficient and the viscosity of the pure cosolvent (Figure 3d). We stress that it is the handbook data of pure cosolvents that is taken to seek the correlations, not the data of solutions. The screening results may be related to the fact that the tridentate coordination of G2 in the Al complex is even stronger than the monodentate coordination of the cosolvent, and the cosolvent can easily desolvate; thereby, the polarity weakly affects the complex structure, and the viscosity affects strongly the diffusion. These results also suggest that even when other cosolvents are used, low viscosity will yield a large diffusion coefficient regardless of its permittivity. Therefore, viscosity is valuable as a screening index for cosolvents when designing high-speed Al plating solutions.

## Conclusions

To understand the complexity of electrochemical deposition phenomena and to enhance the performance, we focus on the physical chemistry of electrolyte solutions, proposing a series of protocols for data-driven material exploration: (i) identification of electrochemically active species structure, (ii) construction of force field parameters specific to the active species structure, (iii) derivation of diffusion coefficients, which is a key factor of electrodeposition, and (iv) data-driven screening of electrodeposition solvents. Specifically, this study outlines design guidelines for glyme-based Al plating electrolytes. Applying the approach of material screening via MD computational data, which has recently garnered attention as a form of data-driven materials discovery, we derived design concepts for room-temperature aluminum plating electrolytes with high ion conductivity. Initially, we hypothesized coordination structures based on previous experimental studies and optimized these structures by using DFT calculations. We demonstrated that G2 dominates the tridentate coordination of cationic aluminum complexes, even in the presence of cosolvent coordination or one-electron reduction. These results led to inferred reduction mechanisms, where the diffusion coefficients of Al complexes were considered as appropriate screening indicators for cosolvents, with values calculated via MD simulations. In general, classical MD is less accurate than DFT–MD but has the advantage of being far less computationally expensive. In addition, it is very essential to make the classical MD parameters of the newly created complex cations in public. Furthermore, we found a strong correlation between viscosity and the physical properties of pure cosolvents and the diffusion coefficients of Al complexes. Thus, this study provides computational insights into the structures and properties that effectively complement experimental data. Future work should focus on evaluating the electrochemical stability of cosolvents as a design factor and extending computational strategies to propose design concepts for different electrolyte solutions.

## Supporting Information Available

Details of the MD simulation, including the procedure, force field parameters, RDF, and MSD, as well as details of the DFT calculation, including desorption energy and Mulliken charge distributions (PDF)
G2 and cosolvent are not shown in the diffusion of the [AlCl_2_]^+^ cationic species for clarity (MP4)
