# Supplementary material for: Computational Screening of Glyme-Based Room-Temperature Aluminum Plating Solutions
Source: ACS Omega. 2025 Jan 10;10(5):4679–85. doi: 10.1021/acsomega.4c09455 (PMC11822509; doi:10.1021/acsomega.4c09455)
Supplement: Supplementary file 1 — ao4c09455_si_001.pdf [file ao4c09455_si_001.pdf]

Supporting Information

Computational Screening of Glyme-Based Room-Temperature Aluminum Plating Solutions

Tomoya Kanno,[1‡] Tsubasa Otsuki,[1‡] Norio Takenaka[1*], and Atsushi Kitada[1*]

[1] Department of Chemical System Engineering, The University of Tokyo, 7-3-1 Hongo, Bunkyo-ku, 113-8656, Tokyo, Japan

*n-takenaka@chemsys.t.u-tokyo.ac.jp

*kitada@chemsys.t.u-tokyo.ac.jp

‡ These authors contributed equally.

· **Details for the MD simulation procedures**

The initial structure was created by Packmol; equilibration was performed three times and the production run was performed, after the system was sufficiently relaxed; the first equilibration was performed with a very small time step and temperature, as the initial structure created by Packmol was unstable (NVT ensemble, temperature 1 K, simulation time 0.0001 ns, time step 0.01 fs), In the second equilibration, the cell size was adjusted by performing NPT-MD (NPT ensemble, temperature 1 K, simulation time 0.0001 ns, time step 0.01 fs). NPT ensemble, temperature 298 K, simulation time 1 ns, time step 1 fs). In the third equilibration, the cell was found to be sufficiently relaxed (NVT ensemble, temperature 298 K, simulation time 0.1 ns, time step 1 fs).The production run (NVT ensemble, temperature 298 K, simulation time 1 ns, time step 1 fs) was performed to obtain the trajectories for deriving the diffusion coefficients.

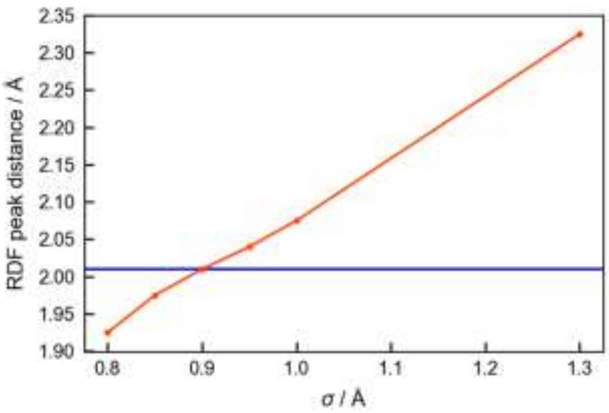

Figure S1 The peak distance of the radial distribution function (RDF) for the Al atom in $[AlCl_2]^+$ with respect to the O atom in G2 (red dots), obtained by varying the Lennard-Jones parameter σ. At σ = 0.9 Å, this distance is 2.01 Å (blue line), corresponding to the value calculated by density functional theory (DFT).

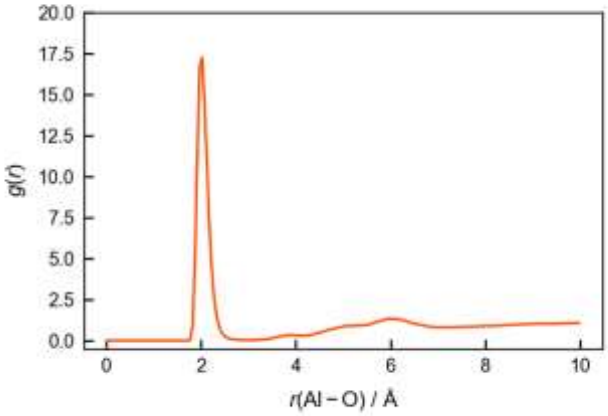

Figure S2 Radial distribution function (RDF; $g(r)$) for the distance r between the Al atom in $[AlCl_2]^+$ and the O atom in G2 when $\sigma = 0.9$ Å, showing a peak at the distance $r = 2.01$ □, the best fit to the DFT results.

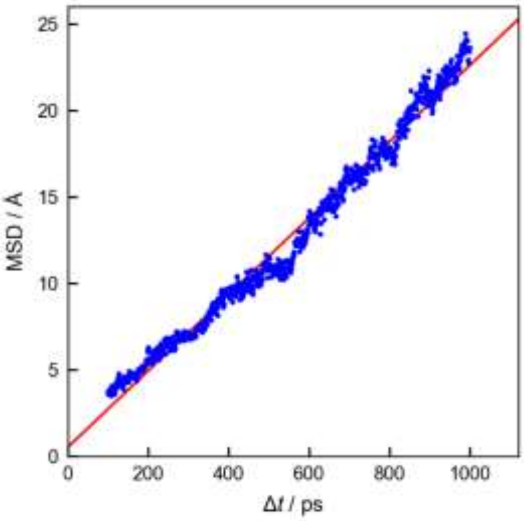

Figure S3 Mean square displacement (MSD) of the Al atom in $[AlCl_2]^+$ (blue dots) and its least-squares fit line (red), obtained by classical molecular dynamics (MD) simulation with the modified force field parameter.

Table S1 Desorption energy of a cosolvent ligand from [Complex C-cosolvent]$^+$ obtained by DFT calculations.

Reaction	Energy/kJ mol^{-1}
[Complex C-EC]$^+$ → [Complex C]$^+$ + EC	12.38
[Complex C-PC]$^+$ → [Complex C]$^+$ + PC	12.12
[Complex C-DMC]$^+$ → [Complex C]$^+$ + DMC	–20.75
[Complex C-$DMSO_2$]$^+$ → [Complex C]$^+$ + $DMSO_2$	18.54
[Complex C-Sul]$^+$ → [Complex C]$^+$ + Sul	22.68
[Complex C-AN]$^+$ → [Complex C]$^+$ + AN	24.33
[Complex C- AcrN]$^+$ → [Complex C]$^+$ + AcrN	22.96

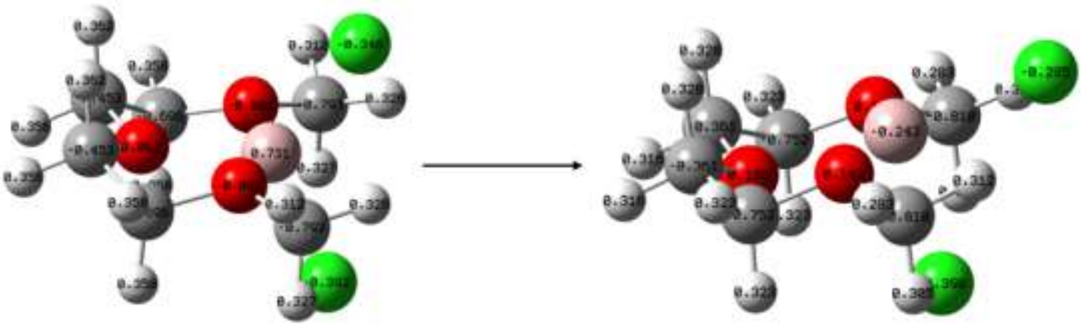

Complex C　　　　Complex D

Figure S4 Optimized structures and Mulliken charge distributions of Complex C and Complex D (white: H, gray: C, red: O, pink: Al, green: Cl).

Table S2 Text data of the force-field parameters of the cationic species $[AlCl_2]^+$.

```
remark goes here
MASS
A2 26.981        0.000
C2 35.45         0.000

BOND
C2-A2     10000      2.00095

ANGLE
C2-A2-C2     10000           180.00

DIHE

IMPROPER

NONBON
  A2          0.900     0.100
  C2          1.679     0.148
```

Table S3. GAFF parameters used in this study		
R_0 (Å)	Cl-Al	2.00095
k_b (kcal mol^{-1} Å^{-2})	Cl-Al	10000
θ_0 (rad)	Cl-Al-Cl	180
k_θ (kcal mol^{-1} rad^{-2})	Cl-Al-Cl	10000
R_{min} (Å)	Al	0.900
	Cl	1.679
ϵ (kcal mol^{-1})	Al	0.100
	Cl	0.148

The total energy is described as follows.

$$E_{total} = \sum_{bonds} k_b(r - R_0)^2 + \sum_{angles} k_\theta(\theta - \theta_0)^2 + \sum_{dihedrals} \frac{1}{2} V_n[1 + \cos(n\phi - \gamma)] + \sum_{i=1}^{N-1} \sum_{j=i+1}^{N} \left[\frac{A_{i,j}}{R_{i,j}^{12}} - \frac{B_{i,j}}{R_{i,j}^{6}} + \frac{q_i q_j}{\epsilon_{i,j} R_{i,j}}\right] \quad \text{(Eq. S1)}$$

Here,

$$R_{min,i,j} = \frac{1}{2}(R_{min,i} + R_{min.j}) \quad \text{(Eq. S2)}$$

$$\epsilon_{i,j} = \sqrt{\epsilon_i \epsilon_j} \quad \text{(Eq. S3)}$$

$$A_{i,j} = \epsilon_{i,j} R_{min,i,j}^{12}, B_{i,j} = 2\epsilon_{i,j} R_{min,i,j}^{6} \quad \text{(Eq. S4)}$$

See also:

[1] J. W. Wang, R. M. Wolf, J. W. Caldwell, P. A. Kollman, D. A. Case, Development and Testing of a General Amber Force Field *J. Comput. Chem.*, **25**, 1157 (2004)

[2] *AMBER 14 reference manual*, Chapter 13.1 https://amber.tkanai-lab.org/TutorialC0/Amber14.pdf Accessed Nov 22, 2024
